# Past, Present, and Future of Rituximab—The World’s First Oncology Monoclonal Antibody Therapy

**DOI:** 10.3389/fonc.2018.00163

**Published:** 2018-06-04

**Authors:** Timothy M. Pierpont, Candice B. Limper, Kristy L. Richards

**Affiliations:** ^1^Richards Laboratory, Department of Biomedical Sciences, Cornell University, Ithaca, NY, United States; ^2^Department of Medicine, Sandra and Edward Meyer Cancer Center, Weill Cornell Medicine, New York, NY, United States

**Keywords:** rituximab, lymphoma, cancer, immunotherapy, monoclonal antibody

## Abstract

Rituximab is a chimeric mouse/human monoclonal antibody (mAb) therapy with binding specificity to CD20. It was the first therapeutic antibody approved for oncology patients and was the top-selling oncology drug for nearly a decade with sales reaching $8.58 billion in 2016. Since its initial approval in 1997, it has improved outcomes in all B-cell malignancies, including diffuse large B-cell lymphoma, follicular lymphoma, and chronic lymphocytic leukemia. Despite widespread use, most mechanistic data have been gathered from *in vitro* studies while the roles of the various response mechanisms in humans are still largely undetermined. Polymorphisms in Fc gamma receptor and complement protein genes have been implicated as potential predictors of differential response to rituximab, but have not yet shown sufficient influence to impact clinical decisions. Unlike most targeted therapies developed today, no known biomarkers to indicate target engagement/tumor response have been identified, aside from reduced tumor burden. The lack of companion biomarkers beyond CD20 itself has made it difficult to predict which patients will respond to any given anti-CD20 antibody. In the past decade, two new anti-CD20 antibodies have been approved: ofatumumab, which binds a distinct epitope of CD20, and obinutuzumab, a mAb derived from rituximab with modifications to the Fc portion and to its glycosylation. Both are fully humanized and have biological activity that is distinct from that of rituximab. In addition to these new anti-CD20 antibodies, another imminent change in targeted lymphoma treatment is the multitude of biosimilars that are becoming available as rituximab’s patent expires. While the widespread use of rituximab itself will likely continue, its biosimilars will increase global access to the therapy. This review discusses current research into mechanisms and potential biomarkers of rituximab response, as well as its biosimilars and the newer CD20 binding mAb therapies. Increased ability to assess the effectiveness of rituximab in an individual patient, along with the availability of alternative anti-CD20 antibodies will likely lead to dramatic changes in how we use CD20 antibodies going forward.

## Introduction

Immunotherapies represent a broad and rapidly growing group of therapies having a substantial impact on cancer outcomes. Their strength is in their potential to activate the immune system to specifically target cancer cells without the broadly damaging side effects of many conventional chemotherapeutics. Monoclonal antibodies (mAbs) were among the initial types of immunotherapy approved for anti-cancer treatment and continue to play a pivotal and growing role in current treatment regimens. Newer therapies have built upon the initial success of mAb therapy. An exciting recent example was the Food and Drug Administration (FDA) approval of two chimeric antigen receptor (CAR)-T cell therapies. These therapies provide high complete remission (CR) rates in patients with otherwise untreatable hematologic malignancies and hold great promise for future advancements. CAR-T cell therapies offer a novel strategy involving *ex vivo* modification and subsequent activation of a patient’s T-cells, but the specificity of the CAR recognition site and subsequent targeting to tumor cells is enabled by mAb technology. Therefore, CAR-T cell therapies and most other immunotherapies rely on mAbs directly or indirectly to target specific antigens on cancer cells. Understanding how best to apply and monitor mAbs and mAb technology is therefore critical for the future success of immuno-oncology.

The first mAb implemented in oncology, and still the most widely used, is the CD20-targeting mAb rituximab. Rituximab is recommended to treat nearly all B-cell non-Hodgkin lymphomas (NHLs). It is most commonly given with cyclophosphamide, doxorubicin, vincristine, and prednisone (R-CHOP), but also with other chemotherapeutic combinations, with small molecule targeted therapies, as a monotherapy, or as maintenance therapy. Despite its widespread use, there is still much uncertainty regarding the mechanism(s) of action of rituximab *in vivo*. We also lack effective predictive biomarkers to identify which patients will respond to rituximab, and when it is given in combination with other chemotherapeutics, we cannot identify patients who are specifically benefiting from its inclusion. This has become more of an issue now that alternative anti-CD20 mAbs have been FDA approved, adding to the impetus to determine when a patient will not respond or has become resistant to rituximab. Furthermore, as rituximab was the first immunotherapeutic used in oncology, it is also the first to have its patent expire, ushering in a swell of competition from biosimilars. Unlike chemical compounds whose efficacy is more easily compared with the originally approved drug, the intrinsic complexity of biologicals is increased by variability that can arise during manufacturing. This complexity, combined with our currently incomplete understanding of the mechanisms behind rituximab efficacy, means that we will need improved methods for determining if these emerging anti-CD20s are as efficacious as the original. This review covers what is known about rituximab’s mechanism(s) of action, activity in various B-cell malignancies, and future directions to optimize the clinical utility of this agent as alternative anti-CD20 antibodies become more prevalent in clinical practice.

### The History of Ritixumab

**Figure 1:**
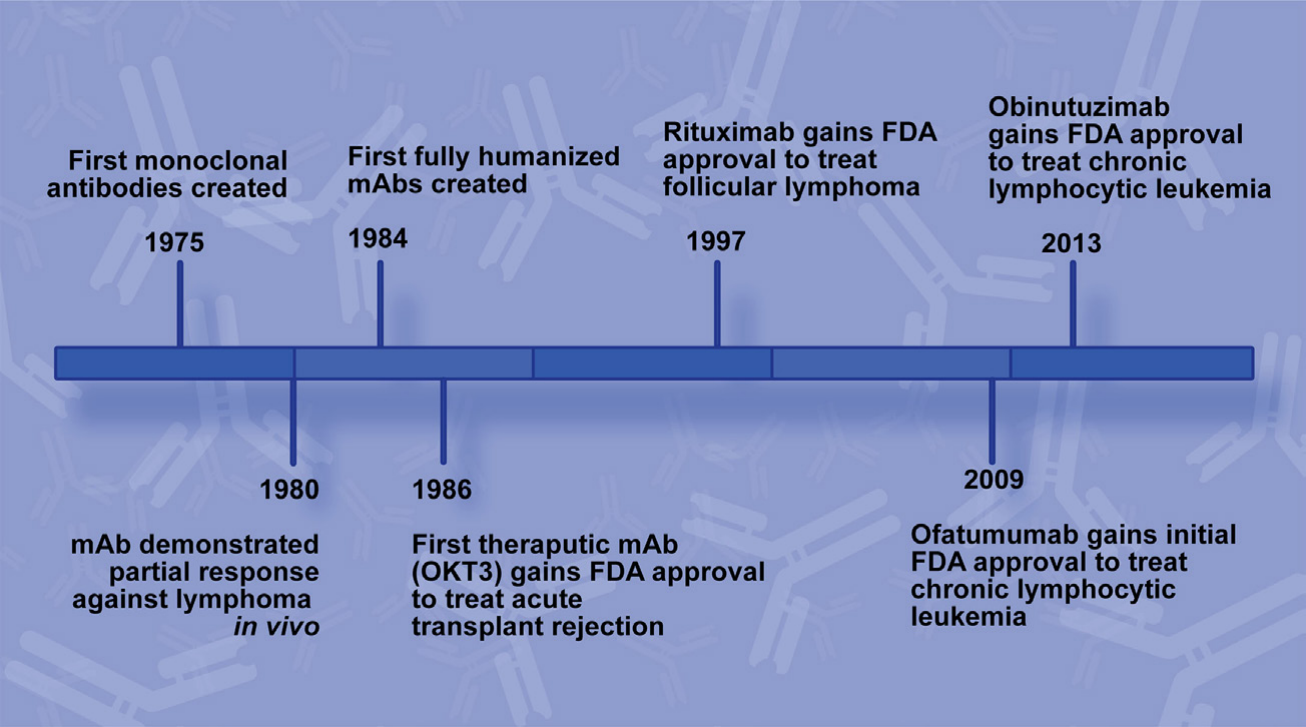
Rituximab development timeline. Key milestones leading to the development of rituximab and additional CD20 monoclonal antibodies (mAbs) for use to treat B-cell non-Hodgkin lymphoma.

While the general concept of immunotherapies has been around for over a century, effective antibody therapies were not feasible before the ability to generate mAbs using continuously growing cell lines (Figure 1). In 1975, Köhler and Milstein generated the first hybridoma cell lines capable of producing mAbs by immunizing mice against sheep red blood cells followed by isolation of B-lymphocytes from the murine spleens and subsequent fusion of those cells with a myeloma cell line (1). The medical and industrial potential of their achievement was quickly realized and has rapidly become a booming biotechnology industry (2).

In 1986, the FDA approved the first mAb for use in a medical application, Muromonab-CD3 (OKT3). OKT3 was developed to treat acute kidney transplant rejection by targeting the CD3 antigen on the T-lymphocytes responsible for the rejection and inducing the death of those cells (3). Oncology mAb therapeutic development is faced with additional challenges, most notably target choice. Optimal targets are universally present on tumor cells but can lead to significant toxicity if their normal cellular counterparts are also targeted.

CD20 is a glycosylated transmembrane phosphoprotein expressed on the surface of developing B-cells, as well as many B-cell malignancies. Because mature plasma cells and B-cell progenitors do not express the protein, depleting B-cells at these intermediate developmental stages generally does not cause permanent side effects. With the limited expression of CD20 among other cell lineages, it was identified as a potential B-cell NHL target for mAb therapy early in the field. Nadler et al. demonstrated a historic proof of principle for mAb immunotherapy in oncology with a preliminary serotheraputic trial in 1980 using an antibody targeted against CD20, designated as Ab 89. The patient, N.B., presented with what was categorized at the time as diffuse poorly differentiated lymphocytic lymphoma that was resistant to standard chemotherapeutics. Although N.B. did not achieve CR, a transient response, measured by a decrease in circulating tumor cells along with an increase in dead circulating tumor cells, provided the first evidence for CD20 as a mAb therapy effective against at least some B-cell lymphomas (4).

Two years before OKT3 approval, another major development in mAb technology was reported. Groups elucidated molecular biology methods for ligating the murine variable region of mAbs with human IgG which generated hybridoma cell lines that produced functional mouse/human chimeric antibodies by retaining the murine variable region but possessing a human Fc region (5, 6). Swapping the murine Fc region for a human one overcame many of the side effects associated with patients developing an adaptive immune response against the therapeutic mAb itself, and it facilitated a more robust immune response against the target due to better binding at the Fc region with human immune effectors. This chimeric technology was the basis for rituximab production, and in 1997 the FDA approved rituximab, brand name Rituxan, for use to treat follicular lymphoma (FL) (7).

Rituximab was created by Ronald Levy for the express purpose of targeting malignant B cells. In 1982, it was made public that his first mAb cancer patient was successfully treated with the mAb, which rapidly lead to the creation of the pharmaceutical company IDEC. Maloney et al. reported the first phase I clinical trials of rituximab, initially named IDEC-C2B8, in 1994, after it had proved effective at killing CD20 expressing cells *in vitro* and in the blood and lymph of macaques (8). Fifteen patients with relapsed NHL were given one of five dosage ranges from 10 to 500 mg/m^2^, and six of those patients experienced tumor regression (8). In 1997, results from a phase I/II trial of 20 patients receiving 125, 250, or 375 mg/m^2^ of rituximab weekly for 4 weeks were published. This study was the basis for FDA approval of rituximab as well as the now standard 375 mg/m^2^ dosage that was used for phase II trials (7). One year later, McLaughlin et al. reported equally impressive benefits of rituximab treatment for patients with relapsed indolent lymphoma, with half of the 166 patients responding to the same four-dose regimen (9).

Due to both its high degree of success as well as its relatively high price, it remained the highest grossing anti-cancer therapeutic through 2016 (10).

### Ritixumab Target: CD20

The hematopoietic stem cell lineage has been well studied. A subset of cells from this hierarchy make the commitment to B-cell development with the transition to pro-B cells within the bone marrow. Following this, they mature into pre-B cells, and then immature B-cells possessing a mature B-cell receptor (BCR) region expressed from VDJ rearranged heavy-, and VJ rearranged light-chain genes capable of recognizing specific antigens. It is at this point that the immature B-cells are negatively selected against for self-reactivity (11). Following this, the remaining immature B-cells move from the bone marrow, maturing into follicular, or marginal zone B cells (11). CD20 expression begins at the early immature B-cell stage, but is not expressed before that point, and is not known to be expressed on other normal cells of the body which has made it a relatively safe and effective anti-cancer target (12).

**Figure 2:**
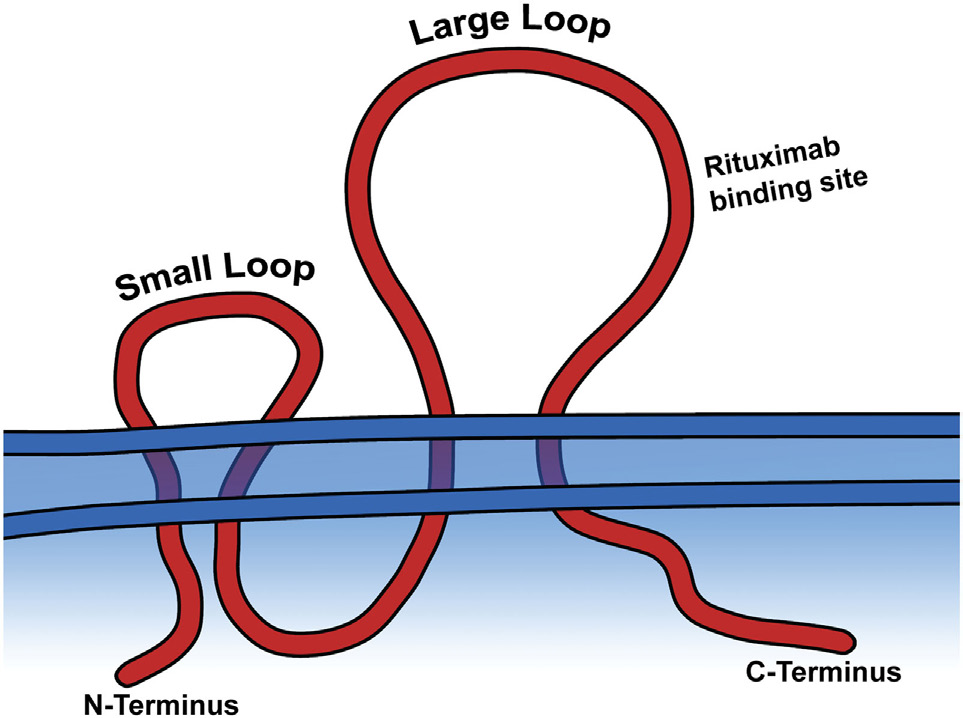
CD20 is a transmembrane protein. The large and small extracellular loops and the general binding site of rituximab are depicted.

CD20 is a tetra-transmembrane protein with an intracellular N- and C-terminal region and two extracellular loops, generally referred to as the small and large loop, and are the portion of the peptide which is targeted by current therapeutic mAbs (Figure 2) (13). Aside from the fact that CD20 is expressed as part of B-cell development, very little is known about its actual biological function. It is known to be involved in store-operated calcium influx, and loss of a cytoplasmic portion of CD20 inhibits activated BCR mediated intake of calcium (14). Also, ectopic expression of CD20 in fibroblasts causes calcium conductance, similar to that of B lymphocytes (14). While it is believed to play a role in B-cell development and activation through calcium influx, it remains unclear if the protein itself is a calcium ion channel, or what other signaling pathways it activates to bring about this function. Despite evidence of its importance in B-cell function, CD20^−/−^ mice harbor no gross phenotype, have normal lifespans, reproductive success, and normal infection susceptibility (15). Surprisingly, even B-cell development was mostly normal in CD20^−/−^ mice, with the main finding being reduced calcium response following IgM ligation (15). These animal model data strengthen the theory that CD20 is involved in calcium intake in B-cells, but the biological significance of that role and the mechanisms employed that facilitate the associated calcium influx remain unsolved.

## Clinical Impact of Rituximab in B-Cell NHL Treatment

Despite the incomplete details on the biological role of CD20, targeting it with rituximab has proven effective for treating a subset of patients in nearly all forms of B-cell NHL. It is frequently given as an initial treatment, either in combination with traditional chemotherapeutics or as a monotherapy. It is also given as maintenance therapy, although benefits of maintenance rituximab (MR) are still unclear for many NHLs. It is rare for B-cell NHLs to be CD20-negative at initial diagnosis, representing only 1–2% of all B-cell lymphomas (16). However, it is more common among B-cell NHL that have relapsed following rituximab treatment, suggesting a selective process toward increased resistance (16). The following section contains current standards of care for the various lymphoma subtypes with historical context from select clinical trials. Current trials and the remaining questions still surrounding the immunotherapy for each specific cancer are also highlighted.

### Diffuse Large B-Cell Lymphoma (DLBCL)

Diffuse large B-cell lymphoma is the most common type of NHL, representing 30–40% of all lymphoma diagnoses in Western countries (17, 18). Since the World Health Organization’s (WHO’s) consensus in 2008, DLBCL has been categorized as germinal center B-cell-like (GCB), activated B-cell-like (ABC), or as unclassifiable lesions which do not fit either profile (19). This classification is based on gene expression profiles that most closely represent the likely B-cell of origin. Along with transcriptional markers, malignancies within these categories share similar genetic aberrations, signaling pathway activation, and clinical outcomes. For example, patients diagnosed with GCB-DLBCL have higher survival, while ABC-DLBCL is more likely to be refractory or relapse. Retrospective studies have shown the benefit of rituximab in both ABC- and GCB-DLBCL, and prospective studies have shown these subtypes to be prognostic for patients treated with either CHOP or R-CHOP (20).

The first phase II single-arm trial for treating DLBCL with rituximab was reported in 2001, studying patients who had aggressive, untreated NHL. Thirty-three patients were included in this trial; the majority of patients (67%) had DLBCL (17). The study showed a 94% overall response rate (ORR) compared with historical CHOP controls (80–90%) and 61% CR compared with historical CHOP controls (44–55%), with only two patients experiencing disease progression by week 24 (21). The increased response rates in this trial were promising, and the use of R-CHOP for these lymphomas demonstrated the feasibility and safety of the regimen in DLBCL treatment.

Approximately 50% of DLBCLs occur in patients over 60, and within that group CR is achieved in only 40–50% of cases when treated with CHOP alone (18, 22). The first phase III trial demonstrating the superiority of R-CHOP in DLBCL, over CHOP alone, was carried out in elderly patients and reported by the GELA group in 2002. This study included patients from 60 to 80 years old who were given CHOP every 3 weeks for eight cycles as tolerated (*n* = 196), or were treated with the same CHOP regimen plus rituximab on day 1 of each cycle (*n* = 202). A CR rate of 76% was achieved with R-CHOP vs. 63% with CHOP (22). A median follow-up of 24 months resulted in an event-free survival (EFS) of 77% for the R-CHOP group and 39% for the CHOP group, reflecting an impressive 42% reduction in risk of events with R-CHOP (22).

An additional study among elderly DLBCL patients was reported in 2006 to look closer at early and late treatment failures and whether MR therapy was beneficial following the successful initial treatment with CHOP or R-CHOP. The study included 415 patients among the four treatment groups with a median follow-up of 3.5 years (23). One important finding from this study was that MR following CHOP resulted in an increased failure-free survival (FFS) compared with only observation following CHOP (23). However, MR following R-CHOP was not significantly different than R-CHOP alone, showing no benefit from MR if rituximab was given during the initial treatment (23).

The phase III MInT trial in 2006 demonstrated the benefits of R-CHOP over CHOP in younger patients, aged 18–60 years, who had a good prognosis. The study involved 824 patients from 18 countries. Individuals who were given R-CHOP had increased EFS (79%) compared with those who received only CHOP (59%) at a median follow-up of 34 months. The R-CHOP group also attained a better three-year OS of 93% compared with 84% with CHOP (24). A 6-year follow-up report by the same group found a 74% EFS among the R-CHOP group compared with 55% with CHOP alone indicating the addition of rituximab to CHOP provides a durable improvement in response for younger DLBCL patients (25).

While GCB and ABC are the more common DLBCL subtypes, primary mediastinal large B-cell lymphoma (PMBCL) is another important subtype of DLBCL specified by the WHO classification of lymphoid malignancies (19). Although it is uncommon, it constitutes approximately 2–3% of all NHL (26). Like all DLBCL, the addition of rituximab has improved both CR and OS over combination chemotherapy alone, and rituximab is now a part of treatment regimens used to treat PMBCL (26).

It is surprising that there is no definitive consensus on optimal dosage of rituximab, despite 20 years of use (27). This is the case for DLBCL, as well as other lymphomas, but efforts are being made to determine if the standard 375 mg/m^2^ is ideal for all patients. Recent findings suggest that dosage may need to be tailored as precision medicine or perhaps increased overall. The SEXIE-R-CHOP-14 trial sought to address the problem that elderly male DLBCL patients had worse outcomes compared with females by increasing the dose of rituximab for elderly males (28). The study showed that increasing the rituximab dose from 375 to 500 mg/m^2^, given every 14 days for six cycles, led to a 32.5% increase in PFS and a 30% increase in OS, although OS increase did not achieve significance (28). Interestingly, these survival rates were slightly better than the elderly female patients treated with 375 mg/m^2^ who were used as the control group in this study which suggests further dosage improvements could have significant impacts on that population as well (28).

In addition, a recent meta-analysis discovered maintenance therapy with rituximab in DLBCL patients improved EFS and PFS, although OS was not significantly improved. However, there was a sex-based difference found in that study as well, with males receiving more benefits from the MR (29).

These findings highlight the need for a better understanding of how rituximab works, its optimal dose and schedule, and the factors that modulate its efficacy, especially between sexes. With a better understanding of those factors, we can optimize rituximab usage by employing a precision medicine strategy.

### Burkitt Lymphoma (BL)

Burkitt lymphoma accounts for 1–5% of adult NHL and is characterized as aggressive lymphoma that is associated with extremely short doubling time caused by MYC dysregulation (17). The disease is usually treated with short-intensive regimens of high-dose cyclophosphamide and methotrexate in combination with vincristine, doxorubicin, and cytarabine, and this has achieved high cure rates in pediatric BL, but a less ideal OS of 64% in adults with the disease (30).

The largest prospective study to date, published in 2014, which spanned from 2002 to 2011 and included 363 patients ranging from 16 to 85 years old demonstrated that the combination immunotherapy was efficacious and feasible, and while the CR rate was not significantly higher than comparable studies without rituximab, OS and PFS were substantially improved (30). Several retrospective studies have attempted to determine rituximab benefits for these patients but were unable to achieve significance (31). However, a recent meta-analysis concluded that there was a significant increase in overall survival when rituximab was given with various chemotherapy regimens compared with chemotherapy alone (31). Also, a 2016 single-arm randomized phase III trial comparing short-intensive chemotherapy alone (*n* = 66) or the same treatment in combination with rituximab (*n* = 70) on BL patients over 18 years of age found that inclusion of rituximab indeed improved 3 years EFS (75 vs. 62%) (32).

Although beneficial, the benefits of rituximab in BL are less clear than for other lymphomas. Indeed, there is some *in vitro* and xenograft model derived evidence that type II anti-CD20 mAb obinutuzumab may work better on BL than rituximab, suggesting mAbs of CD20 with differential binding to either CD20 or immune effectors, may lead to better results for some lymphomas (33). This would be clinically important, but could also elucidate the mechanism(s) of therapeutic response of rituximab and other anti-CD20 mAbs.

### Mantle Cell Lymphoma (MCL)

Mantle cell lymphoma is a moderately aggressive lymphoma that comprises 2–4% of all NHL and has a median OS of 3–5 years (17). MCL is technically classified as an indolent lymphoma; but it usually has an aggressive clinical course and is incurable, despite an initial response to either dose-intense chemotherapy or combination therapy (34). Although rituximab has proven beneficial as a maintenance therapy, R-CHOP achieves a relatively short median PFS of 16–17 months (17, 34, 35). Several chemotherapeutic regimens are recommended to treat MCL, including bendamustine, CHOP, high-dose cytarabine, or fludarabine-based regimens (34). Rituximab is also generally used in combination, despite few studies directly evaluating the efficacy of rituximab in treating MCL, and retrospective analyses have concluded addition of the immunotherapeutic does indeed improve OS (34, 36).

Rituximab maintenance has demonstrated an OS benefit in a phase III randomized MCL clinical trial, which has not been shown in other lymphoma subtypes (37). The study compared MR (*n* = 120) after autologous stem cell to observation only (*n* = 120) and found a 4-year PFS of 83 and 61%, respectively (37). The MR group also had a significantly increased OS (37). Interestingly, retreatment with rituximab when molecular relapse occurs has also proven a successful strategy to regain molecular remission status, and likely to prolong clinical remission time (38, 39). This could provide a strategy for more cost-effective maintenance of remission in MCL.

### Indolent Lymphomas

Unlike the more aggressive NHLs, indolent NHLs progress more slowly. Following diagnosis, the disease can be treated immediately or treatment may be delayed until symptoms appear. Because of this, indolent lymphomas have a longer median survival; but while they progress slowly and often respond to initial treatment, they also relapse and ultimately tend to be incurable. Rituximab monotherapies and rituximab in combination with chemotherapeutics have had a significant impact on the survival of patients with these lymphomas.

One important question that remains to be fully answered is the benefit of maintenance therapy in treating indolent lymphomas, which is where rituximab and other mAbs may play a pivotal role in increasing FFS or OS since they can be more safely given long term due to their lower toxicity (40). Despite extensive research, it remains uncertain how helpful maintenance therapies are for most indolent lymphomas. Outlined below are important historical trials, as well as recent advances using rituximab as part of initial therapies and MR on specific indolent NHLs.

### Follicular Lymphoma

Follicular lymphoma arises from malignant transformation of follicle center B-cells and accounts for approximately 20% of adult NHLs in the Western countries (17). FL has an indolent clinical course with an average OS rate of 73% at 10 years with modern treatments, but the majority of cases are ultimately still incurable (41). The median age at diagnosis of FL is 55–60 years old, and it occurs slightly more frequently in females (42).

Follicular lymphoma was the first cancer for which the FDA approved rituximab use. The milestone phase II study evaluated 37 FL patients with low-grade relapsed disease and treated them with four weekly doses of 375 mg/m^2^ as a monotherapy (43). Clinical remission was achieved in 17 patients (46% response rate, 3 patients achieved CR) with a median time to progression of 10.2 months among those responders (43). The results of this study showed not only the safety and feasibility of treating FL with rituximab; it demonstrated clear efficacy which led to its approval. A phase II/III multicenter trial published in 1998 included 166 patients with recurrent indolent FL patients from 31 centers (9). Rituximab was given as a monotherapy on the same dosage schedule as the 1997 study and again achieved a 48% response rate (6% achieved CR) with a median time to progression of approximately 12 months among responders (9). The remission rates of these studies were comparable to response rates achieved by standard chemotherapeutics (9). In 1999, another milestone study was published which was aimed at testing the safety and feasibility of combination CHOP and rituximab (44). The study included 40 indolent NHL patients given R-CHOP and achieved an impressive 95% ORR (55% CR) and helped solidify R-CHOP and other rituximab combination therapies as the current standard of care for most CD20 expressing NHLs. A recently published phase II trial composed of 66 FL patients determined lenalidomide in combination with rituximab may be a reasonable R-CHOP alternative as it yielded similar CR and PFS rates current therapies with low toxicity (45). This is being evaluated in the phase III RELEVANCE study, and interim results have not demonstrated superiority of either regimen (46). Half of all FL patients are 60 or above, and treatment choices in these groups can be more difficult due to overall health and comorbidities. Still, the low toxic side effects of rituximab compared with chemotherapies indicate mAb as a safe and effective treatment in elderly FL patients both in combination or often as a monotherapy (47).

Because of its indolent nature, FL often does not require immediate treatment. There is uncertainty surrounding what the best treatment is, if any, during asymptomatic periods following diagnosis. This is a point of contention for both the time before initial treatment, as well as optional maintenance therapy following remission, with the alternative option being “watchful waiting” in which treatment begins only once symptoms or impending organ failure occurs. In retrospective studies and several clinical trials, there was no significant survival benefit to starting treatment early compared with watchful waiting (48). Likewise, maintenance strategies are similarly not well established to have an overall survival benefit. The PRIMA study of MR enrolled 1,217 patients and, following induction therapy, randomized them into groups receiving either observation or 2-year MR (375 mg/m^2^ rituximab every 8 weeks) (49). In a 6-year follow-up report, the group concluded a significant benefit to PFS, but not OS (50). Another phase III trial published in 2014 enlisted 379 patients with low-tumor-burden FL for either watchful waiting, rituximab induction (375 mg/m^2^ weekly for 4 weeks), or rituximab induction followed by MR consisting of 12 additional infusions given every 2 months over 2 years (48). The key endpoint for this study was time until the disease progressed to the point of needing treatment. Within the watchful waiting group, only 46% of patients had not yet required treatment by 3 years while 78% of patients within the rituximab induction group and 88% of patients within rituximab induction plus MR group did not require treatment by the same timepoint (48). Interestingly, quality of life metrics were significantly higher in the group receiving MR than in the other two groups. These data argue that rituximab may significantly delay the need for chemotherapy in FL, and given the relatively low toxicity, could be considered as initial therapy in this group of patients. Since both induction rituximab and induction followed by MR produce similar response rates, it is unclear what mechanisms provide the durable remission considering that continued dosage had a minimal additional benefit in disease response. Although the immune effectors of rituximab are not associated with memory, there is growing evidence to support memory–natural killer (NK) cells with cytotoxic capacities and these cells, or possibly some unknown effector mechanism, may be responsible for the durable delayed disease progression (51). A recent meta-analysis found MR may also provide improved overall survival in all FL patients based on findings across seven trials including 2,315 patients, although OS benefit to the subgroup of patients receiving R-chemo in the first-line setting was not demonstrated. These findings have not been replicated in phase III trials, and importantly did not include patients treated with bendamustine, which has subsequently become a standard frontline regimen for FL (52).

### Marginal Zone Lymphoma (MZL)

Marginal zone lymphoma is an indolent lymphoma that comprises 5–10% of all NHL (17). Randomized trials are lacking to demonstrate the efficacy of rituximab in this lymphoma subtype specifically, but rituximab is usually included in treatment regimens, and single-agent activity has been demonstrated (53, 54). There are three main categories of MZL, with the majority being classified as extranodal marginal zone B-cell lymphoma of mucosa-associated lymphoid tissue (MALT). These cancers are often associated with an infectious agent (e.g., gastric MALT is associated with *H. pylori*) and can sometimes be eradicated with successful treatment of the underlying infection. If further treatment is needed for localized disease, radiation treatment often leads to long-term remissions. Systemic treatment for widespread disease consists of a combination of chemotherapy (e.g., bendamustine or chlorambucil) and rituximab, or with either reagent alone. Efforts are being made to identify the ideal treatment based on a prognostic index in the post-rituximab era (55).

Splenic MZL is a rarer form of MZL. There are no standardized treatments for splenic MZL due to a lack of randomized trials. However, rituximab or rituximab with chemotherapy is often used (56). Unlike reported for DLBCL and FL, rituximab combined with chemotherapy has not yet been demonstrated to improve survival, while rituximab monotherapy is reported to achieve a 69% 7-year PFS (56).

Nodal MZL is another indolent lymphoma that is thus far incurable but has a 5-year survival rate of 70–90% with current treatments (57). This disease also has no standard treatment, but when localized is usually treated with radiotherapy, while high tumor burden disseminated disease is treated with rituximab in combination with various chemotherapy regimens including bendamustine, fludarabine, or fludarabine with cyclophosphamide (57).

### Lymphoplasmacytic Lymphoma (LPL)

Lymphoplasmacytic lymphoma follows an indolent clinical path and is incurable but rare. Due to its indolent nature, it has a median survival of 5–10 years in symptomatic patients (58, 59). The disease comprises cells most similar to those intermediate between small lymphocytes and true plasma cells, with features of both, including secretion of an IgM paraprotein (17). Multiple chemotherapy treatment regimens exist, including those based on alkylators (e.g., bendamustine), proteasome inhibitors (e.g., bortezomib), nucleoside analogs (e.g., fludarabine), or mAb ibrutinib. Since LPL is CD20-positive (unlike plasma cells) and rituximab has shown activity as a single agent in this disease (60), rituximab is often added in combination with chemotherapy regimens (61, 62).

The vast majority of cases of LPL are classified as Waldenstrom macroglobulinemia, which has pathophysiology in part determined by the two key mutations, such as *MYD88*^L265P^ and *CXCR4*^WHIM^. While the disease is considered incurable, asymptomatic patients are not treated until symptoms appear, like other indolent lymphomas. There is no single recommended treatment for this disease, but it is treated with combination regimens including rituximab and fludarabine, oral cyclophosphamide with cladribine or fludarabine, as well as fludarabine, cyclophosphamide, and rituximab (FCR) as the first-line therapies for the disease (59). Since rituximab can cause an IgM flare, it should not be used until the IgM paraprotein levels are below 4,000 mg/dL. MR in rituximab-responsive patients was shown to improve OS in an observational study, but no randomized studies have proven the effectiveness of this strategy (63).

### Hairy Cell Leukemia (HCL)

Hairy cell leukemia is a lymphoma of mature B-cell origin, despite its name. It is a rare chronic disease with a good prognosis, with a small percentage (~10%) not requiring immediate treatment but instead observation until treatment becomes necessary (64). It is regarded as one of the few cancers that were once generally fatal but is now almost always curable or maintainable, usually allowing patients to reach normal life expectancy (65).

The disease is effectively treated with nucleoside analogs, but patients relapse. A recent phase II study found that cladribine followed by rituximab achieved a durable remission of nearly 100% 5-year FFS in HCL patients (66).

### Chronic Lymphocytic Leukemia (CLL)

Chronic lymphocytic leukemia, also referred to as small lymphocytic lymphoma depending on where the primary presentation of the disease occurs, which can be in the peripheral blood, bone marrow, or solid lymphoid organs. Despite the different names and primary locations, the two diseases comprise the same type of lymphocyte and share similar pathogenesis and prognosis (67). The disease is indolent with a relatively high median survival. Importantly, CD20 expression in CLL patients tends to be lower compared with other B-cell lymphomas (68). Although rituximab does have clinical relevance in CLL, it is thought this lower expression of CD20 may be why the mAb is not as beneficial in these lymphomas and is the reason newer, possibly more potent, anti-CD20 drugs were first tested in CLL (68, 69).

In CLL patients who are young and fit and lack deletion of 17p or TP53, current treatment guidelines recommend chemotherapy, commonly fludarabine, cyclophosphamide in combination with rituximab (FCR) as initial treatment based on the proven effectiveness of rituximab from several clinical trials (70). A recent Canadian study confirmed the tangible benefits in CLL by evaluating patients treated in the pre- and post-rituximab era (71).

The currently ongoing FLAIR phase III trial includes 754 CLL patients given either the current standard of care FCR, ibrutinib plus rituximab, ibrutinib plus venetoclax, or ibrutinib alone, potentially eliminating the need for more harmful chemotherapeutics in favor of more targeted therapeutics as the new standard of care (70). This study should also assess the benefit of the addition of rituximab to small molecule targeted therapies (in this case ibrutinib), which has been relatively understudied.

### Rituximab Depletion of Non-Malignant B Cells to Treat Autoimmune Diseases

Because rituximab depletes normal B cells, it has also been effective in treating a wide variety of autoimmune diseases by reducing the adaptive immune response against self. The FDA approved it for treating rheumatoid arthritis (RA) in 2006, and it has shown promise in treating some other autoimmune disease as well (72). Both case reports and meta-analyses indicate rituximab helps alleviate symptoms, even in refractory patients, of pemphigus (73), pemphigoid (74), myasthenia gravis (75), and neuromyelitis optica (76). However, despite successful clinical trials for RA, not all autoimmune diseases respond as well to rituximab. Systemic lupus is one unfortunate example where recent randomized, double-blind phase II/III trials found no significant benefit of adding rituximab to the standard of care (77, 78).

The increasing use of rituximab to treat RA since 2004 has told us a lot about normal B cells’ response to the mAb (79). Four weekly doses of 375 mg/m^2^ rituximab depletes B cells from the peripheral blood for approximately 6 months in RA patients, although response duration varies between individuals (80). Surprisingly, B-cell depletion is well tolerated among most patients and has limited negative health effects. Increased risk of infections and late-onset neutropenia are two of the most common problems, while reduced vaccine efficacy is also thought to be an issue (81). The vast majority of information on rituximab response comes from monitoring of peripheral blood. However, there is only a modest drop in antibody production in RA patients treated with rituximab, suggesting incomplete depletion of B cells in the spleen, lymph nodes, and bone marrow (81).

## Shortfalls of Rituximab and Alternatives

Although rituximab as a monotherapy, or in combination with chemotherapeutics, has greatly improved the prognosis of all B-cell NHL, there are still many cases in which it fails. In the case of DLBCL, 30–50% of patients are not cured by R-CHOP, with about 20% being initially refractory and another 30% relapsing after CR (82). The majority of indolent NHLs will eventually relapse and are incurable. This high rate of failure has spurred on the search for improved methods of treating refractory or relapsed patients, emphasizing the need for new biomarkers that identify those who will, and those who will not, be effectively treated by rituximab-based regimens (83).

### Subcutaneous Rituximab

A relatively recent development in rituximab therapy was the FDA approval of a subcutaneous formulation of the mAb which combines it with recombinant human hyaluronidase. Recombinant human hyaluronidase is used to increase the dispersion and absorption of molecules and thus allow very small, highly concentrated volumes to be injected subcutaneously while retaining efficacy (84). In 2014 a randomized phase III study, SABRINA, evaluated the pharmacokinetics (PK) and safety of subcutaneous rituximab in FL. The study compared 48 patients who received subcutaneous rituximab to 54 who received intravenous rituximab and found that subcutaneous delivery was non-inferior (85). The subcutaneous delivery was also preferred by nearly all patients, and the benefits include less time in the clinic with anticipated reduced workloads for clinical staff, lower health-care cost, and increased accessibility of rituximab therapy (86). Following the 2014 study, similar trials have found subcutaneous rituximab to be non-inferior in treating CLL and DLBCL as well (84). The subcutaneous formulation was approved by the FDA in 2017 to treat FL, DLBCL, and CLL.

### Radiolabeled and Toxin Conjugated Anti-CD20

Rituximab is a powerful antitumor reagent with relatively low side effects but, as discussed, its mechanisms of action are still not well understood, hampering efforts toward further improving patient survival. One method of modifying rituximab and other anti-CD20 mAbs is by conjugation of a radiolabel or cytotoxic drugs, delivering the toxic payload directly to the targeted B-cell malignancies.

Radiolabeled anti-CD20 antibody tositumomab (Bexxar) and ibritumomab tiuxetan (Zevalin) have produced higher CR rates compared with unlabeled mAbs, and one course is approximately as effective as six to eight cycles of combination chemotherapy (87). Both were approved by the FDA in 2002 and 2003, respectively, to treat several relapsed or rituximab-refractory NHL subtypes. Logistical obstacles have prevented them from being widely used, and despite the success of these first-generation radiolabeled mAbs they both suffered from poor sales, which ultimately lead to Bexxar being pulled from the market (88).

Ritixumab conjugated to doxorubicin is one example of toxin conjugated anti-CD20 therapy. This strategy has been further modified to improve efficacy, including attempts to generate reduction-sensitive micellar nanoparticles for better delivery, although neither these nor any similar anti-CD20 conjugate with toxins, have been approved by the FDA to date (89).

### Additional CD20 mAbs for Lymphoma

**Figure 3:**
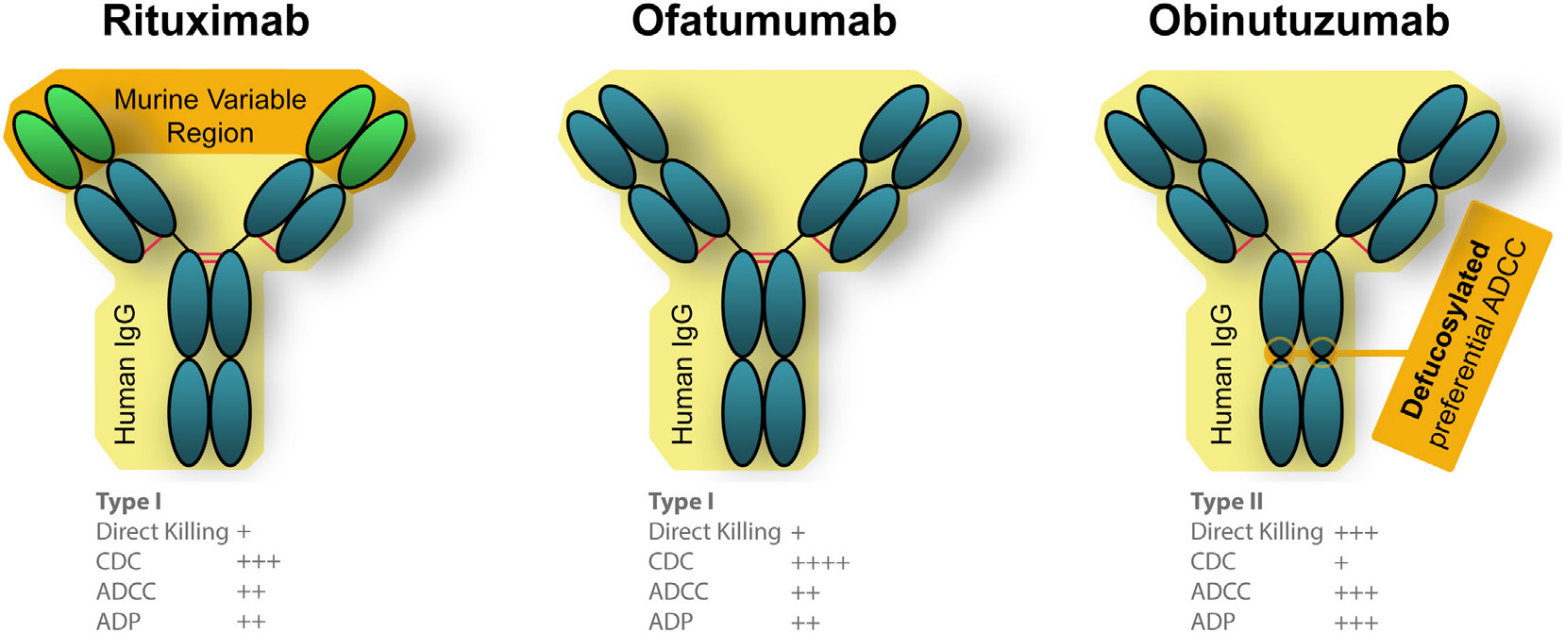
Engineered differences between Food and Drug Administration approved anti-CD20 monoclonal antibodies (mAbs). Rituximab is a chimeric mAb that is partially humanized, that has a human Fc portion but retains the murine variable region which recognizes CD20. Both ofatumumab and obinutuzumab are fully humanized mAbs, which reduces unintended immune responses against the therapies. Ofatumumab also has a glycoengineered Fc region which results in better binding with immune effector cells (106, 190).

Several additional therapeutic anti-CD20 mAbs have been generated since the advent of rituximab. Each features “next generation” modifications: an alternate binding epitope, additional humanization, altered glycosylation, or another combination of modifications (Figure 3). Two have already been approved by the FDA, ofatumumab, and obinutuzumab, while many others are in various phases of development of both type I and type II anti-CD20 mAbs. Type I mAbs translocate CD20 to lipid rafts, preferentially activate complement-dependent cytotoxicity (CDC) and antibody-dependent cell-mediated cytotoxicity (ADCC), have a weak homotypic adhesion, and have a caspase-dependent apoptosis induction (90). On the other hand, type II mAbs do not rearrange CD20 to lipid rafts, have a higher affinity toward ADCC induced death, and caspase-independent induced by a lysosome-mediated mechanism (90).

### Ofatumumab

Ofatumumab (trade name Arzerra) became the first fully humanized mAb targeted to CD20 to gain initial approval for anti-cancer therapies by the FDA in 2009, and full approval in 2014. This mAb binds to a different epitope than rituximab, which binds the large extracellular loop of CD20 (91). Ofatumumab, on the other hand, can bind both the small and large extracellular loop of CD20 (91). This unique binding, which is more proximal to the cell membrane, is suspected to be the source of increased CDC activity compared with rituximab (92). Being fully humanized, ofatumumab should cause less anaphylaxis, and a recently released case study reported it was successfully administered without reaction to a patient who had previously presented with anaphylaxis in response to rituximab (93).

Ofatumumab is approved to treat CLL that is refractory to fludarabine and alemtuzumab therapies (94). The study included 59 patients refractory to fludarabine and alemtuzumab and 79 patients with bulky lymphadenopathy refractory to fludarabine alone (95). Patients were given an initial 300 mg of ofatumumab dose followed by 11 additional doses at 2,000 mg over 24 weeks (95). The ORRs were 58 and 47%, respectively, among the two groups with an OS of 13.7 and 15.4 months, respectively (95). While this study showed ofatumumab to be efficacious in refractory CLL, it was not directly compared with rituximab. It is worth noting that a retrospective follow-up study examined response based on prior rituximab exposure and found ofatumumab achieved an ORR of 44% in patients who were refractory to rituximab (96). A 2001 rituximab study found doses of 2,250 mg/m^2^ achieved a 75% overall response, making the higher doses of ofatumumab a confounding factor for comparison of efficacy between the two mAbs (97). A 2015 phase II trial treated 49 indolent NHL patients with bendamustine and ofatumumab and found the ORR comparable to historical treatments with bendamustine and rituximab (98). A 2017 study by the Alliance found PFS was comparable between ofatumumab with bendamustine (OB) and historical rituximab with bendamustine in previously untreated FL, despite an initially improved CR with OB (99). Given conflicting reports of increased benefits, additional randomized phase III trials and more biologically representative *in vitro* assays are needed to fully assess the differences in efficacy between these CD20 mAbs.

### Obinutuzumab

In 2013, obinutuzumab (trade name Gazvya) became the first glycoengineered antibody approved in the US as the next generation of anti-CD20 mAb for cancer treatment. The glycoengineering is accomplished by overexpressing two glycosylation enzymes, MGAT III and Golgi mannosidase II which resulted in antibodies that are mostly non-core-fucosylated and possess unique properties distinct form regular IgG1 (100). mAbs of this particular subisotype are also referred to as IgG(1E5) (100). These modifications create better binding of effector immune cells and a more efficacious response compared with rituximab, although the clinical benefits have been variable. A phase II trial which tested obinutuzumab in combination with chlorambucil for previously untreated CLL patients found similar response rates compared with rituximab and ofatumumab in similar patient groups (101). FDA approval was based on a subsequent phase III trial (102). It is important to note that obinutuzumab (and ofatumumab, as discussed above) were given at substantially higher doses compared with rituximab, making a direct comparison of efficacy difficult (95, 101).

In the phase III GOYA study of DLBCL patients who compared G-CHOP (*n* = 706) and R-CHOP (*n* = 712) followed out to a median observation of 29 months, Vitolo et al. found no improvement in PFS after treatment with obinutuzumab vs. rituximab plus CHOP (103). A recent phase III trial treated FL patients with either R-CHOP (*n* = 601) or G-CHOP (*n* = 601) and followed their progression for a median of 34.5 months (104). Unlike the similarly powered DLBCL study, this group found that G-CHOP with maintenance therapy provided an increased PFS (104). In February 2016, obinutuzumab was approved to treat patients with FL who relapsed or have refractory disease to any rituximab-containing regimen (105).

Unlike in DLCBCL, but similar to FL, recent CLL clinical trials comparing G-CHOP to R-CHOP appear to show a better response to obinutuzumab combined with CHOP rather than rituximab (106). Although the data are preliminary and based on higher doses of mAbs given for both obinutuzumab and ofatumumab, it suggests different CD20 antibodies may work better for specific lymphomas, and clinical trials for each mAb may result in more personalized medicines. However, better methods for rapid screening of efficacy of specific anti-CD20 mAbs against an individual’s lymphoma are needed to achieve effective precision medicine that would be clinically most useful.

### Ublituximab

Ublituximab is a type I glycoengineered anti-CD20 mAb that binds to an epitope unique from rituximab, ofatumumab, or obinutuzumab and contains a low-fucose Fc region that facilitates enhanced ADCC activity *in vitro* (107). A recent phase I/II trial included 45 patients with relapsed or refractory CLL who were treated with a combination of ublituximab and ibrutinib (107). The treatment achieved an ORR of 88%, with a 5% CR but the durability of the response is not yet known, and while the safety and feasibility of ublituximab have been established, an ongoing phase III study will determine if the anti-CD20 increases the efficacy above ibrutinib monotherapy (107).

## Mechanisms of Rituximab Response

**Figure 4:**
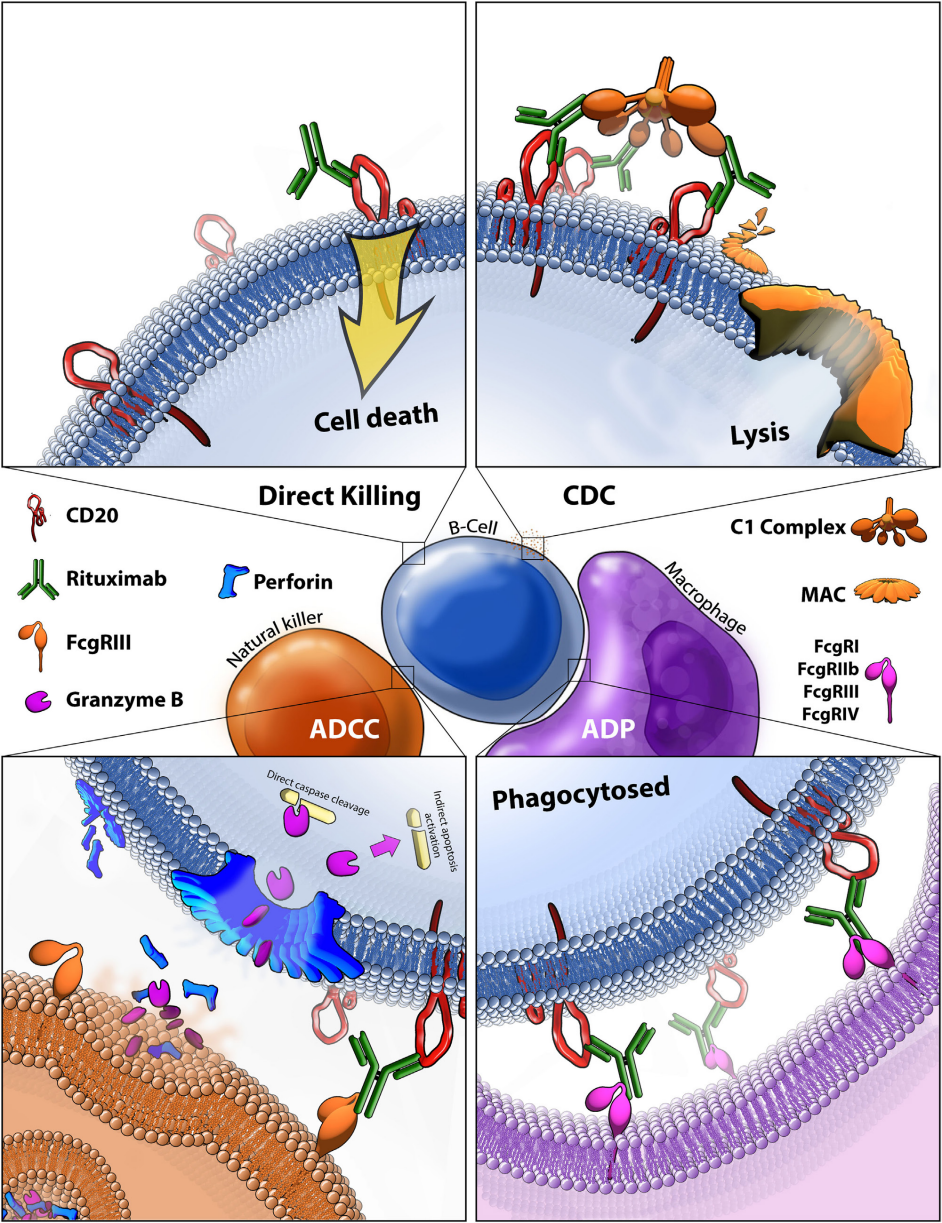
Rituximab-mediated cell killing of CD20 expressing B-cells. (Top left) Binding of rituximab to CD20 can directly trigger apoptosis through both caspase-dependent and -independent mechanisms that are still not fully characterized. (Top right) Bound rituximab can recruit the C1 complex triggering the classical complement cascade which leads to insertion of the membrane attack complex (MAC) and ultimately leads to cell lysis, also known as complement-dependent cytotoxicity (CDC). (Bottom left) Bound rituximab can recruit natural killer cells *via* recognition by the FcγRIII leading to antibody-dependent cell-mediated cytotoxicity (ADCC). This facilitates release of perforin, which assembles into membrane compromising pores in the target cell, and granzyme B, which enters the target cell and triggers apoptosis by cleaving caspases and potentially by other methods. (Bottom right) Macrophages recognize CD20 bound rituximab through various Fcγ receptors which leads to antibody-dependent phagocytosis (ADP) of the target cell.

The binding of rituximab to CD20 facilitates cell death in four main ways, three of which rely on recruiting effector mechanisms from the patient’s own immune system. Because of this reliance on the human immune system (HIS) to mediate antitumor effects, the exact *in vivo* mechanisms remain challenging to study. Based on a combination of *in vivo, ex vivo*, and *in vitro* work, we know that rituximab-mediated killing occurs by triggered cell death *via* binding of rituximab to CD20, CDC, ADCC, and antibody-dependent phagocytosis (ADP) (Figure 4). One major barrier to fully understanding the mechanisms of the immune system in immunotherapies is the lack of an ideal animal model. Because rituximab is targeted against human CD20, it can be evaluated in immunocompromised mice xenografted with human lymphoma, but these mice do not possess human immune cells or human complement proteins. Much work is being done to better model human NK cells in mice to provide more biologically relevant animal models, mainly through the development of HIS mice (108). One major issue is achieving normal NK cell development in a murine body. Recent findings suggest knocking in human *SIRPA* and *IL15* to replace the wild-type copies in HIS mice resulted in normal tissue distribution circulation of NK cells. Furthermore, the NK cells in these HIS mice can facilitate ADCC, providing a crucial next step toward research tools for understanding the role of rituximab-mediated ADCC *in vivo* (109). Still, the complexities of rituximab response are further complicated by potential competition and synergy between all other immune effector responses, including direct cell killing.

### Direct Signaling Induced Cell Death

Although presumed to have limited contribution to the *in vivo* antitumor effects of rituximab, many *in vitro* studies have demonstrated that binding of the mAb can trigger cell death without immune system effector mechanisms. Two main pathways for this direct cell killing have been identified which are caspase-dependent, and -independent (Figure 4, top left). Surprisingly, despite over 30 years of intensive study, no CD20 ligand has been discovered, making it difficult to predict and understand how anti-CD20 binding alone might trigger cell death. Rituximab binding to CD20 causes rearrangement of lipid rafts and alters CD20 localization; defining it as a type I CD20 antibody (110). It is not entirely known how this rearrangement triggers cell death, it is known that the process is src family kinase-dependent and results in caspase-mediated apoptosis (111). Although relatively little is known about the molecular pathways of cell death *in vivo*, Akt, ERK1/2, NF-κB, and p38 MAPK are pathways shown to be involved in rituximab-mediated apoptosis (112). Ivanov et al. found that type II CD20 antibodies primarily induce cell death without lipid raft formation, through actin reorganization leading to lysosome-mediated cell death, independent of caspase pathways (113). No direct evidence of human *in vivo* killing by this mechanism has been found, but one compelling study demonstrated a reduction in CNS lymphoma after rituximab was injected directly into the cerebrospinal fluid, where limited immune responses are available, arguing for a direct cell killing mechanism (114).

### Complement-Dependent Cytotoxicity

Complement-dependent cytotoxicity is mediated by the classical pathway of the complement system. The C1 complex binds to rituximab opsonized cells and triggers the complement cascade which results in the insertion of the membrane attack complex (MAC) into the target cell membrane, thus compromising the membrane and triggering cell lysis (Figure 4, top right). CDC is known to play some role in the *in vivo* killing of B-cell malignancies, potentially having the largest effect on circulating tumor cells and contributing to the recruitment of immune cells, although the true extent of its contribution to response is still unknown (115).

There is evidence that CDC is not as effective as ADCC *in vivo* and an effective CDC response may have a negative overall impact on rituximab efficacy as both processes compete for access to the bound mAb (116). Different anti-CD20 antibodies have different propensities to activate CDC (115). Studies have also shown a competitive relationship between ADCC and CDC *in vitro* (117).

Other studies that suggest the importance of CDC *in vivo* centers around the frequent observation of complement-regulatory proteins CD55 and CD59 were expressed on circulating tumor cells (118). When tested *in vitro*, high expression of these proteins were associated with increased resistance to rituximab, but their neutralization overcame that resistance (118). In addition, one study utilizing sera collected from CLL patients demonstrated patients were more frequently deficient in C1q, C3, and C4 complement proteins and that their sera was more readily exhausted of complement activity following anti-CD20 mAb treatment, resulting in lowered CDC activity (119). On the other hand, in some mouse studies with genetic deficiencies for either FcR common γ chain-deficient or complement components C3, C4, or C1q, it was found that CDC does not play a role in the killing of circulating tumor cells utilizing murine anti-CD20s to target murine lymphoma (120, 121). Therefore, the impact of CDC on rituximab-mediated anti-cancer effects *in vivo* is still not fully defined, interactions between ADCC and ADP with CDC have yet to be addressed, and additional *in vitro* methods for characterizing those interactions need to be further developed.

### Antibody-Dependent Cell-Mediated Cytotoxicity

Antibody-dependent cell-mediated cytotoxicity is thought to be a significant contributor to the *in vivo* antitumor activity of rituximab. Binding of the variable region of the mAb to CD20 facilitates the binding its Fc region to FcγRIII receptors on NK cells, thus leading to the formation of the immune-synapse that consists of the region where the two cells make contact (Figure 4, bottom left). This binding triggers a response in cytotoxic NK cells to release granules containing perforin, which self-compiles in a Ca^2+^-dependent manner into a non-selective pore which embeds into and permeabilizes the membrane (122). The NK cells also release granzyme B at the immune-synapse, which infiltrates the permeabilized membrane of the target cell and induces programmed cell death, through various ways including caspase-dependent mechanisms, having the ability to cleave caspase 3, 6, 7, 8, 9, and 10 directly, as well as activate caspase 2, 6, and 9 indirectly (123).

Detecting and quantifying rituximab-mediated ADCC *in vivo* is challenging for the same reasons as CDC, in that it largely requires a functional HIS and therefore makes animal model data more difficult to interpret. Nonetheless, a mouse study demonstrated FcγRs were necessary and sufficient for anti-CD20 depletion of various cancers in both xenografted and syngeneic models (124).

Quantifying ADCC *in vitro* has proven challenging due to the necessity of combining NK effectors and target cancer cells into the final reaction which makes it difficult to separate NK cell death from that of the target cells. Originally, ^51^chromium (^51^Cr) was used to measure lysis by NK cells by first having the target cells uptake the ^51^Cr, then combining the cells and measuring the amount of ^51^Cr released into the supernatant, thus indirectly measuring the percentage of cells lysed. Similarly, fluorescence assays were developed using calcein-acetoxymethyl which is taken in and cleaved by living cells to generate a hydrophilic fluorescent molecule that is trapped within intact membranes (125). Both methods are indirect, can be influenced by factors unrelated to actual cell death, and are often hard to reproduce which makes them difficult to use for highly sensitive measurements (126). A luciferase assay was recently published as an alternative method to the release assays by creating novel effector cells expressing variants of FcγRIIIa believed to impact ADCC activity (127). This also relies on the indirect measurement of cell killing and requires using specific effector cell lines (127). Recently, a flow cytometry-based assay was published using is a small molecule, CFSE, that binds to proteins of live cells thus labeling target cells fluorescent green before combining in the ADCC assay and then directly measuring the percentage of dead target cells *via* flow cytometry. This proved more accurate than release assays and required only 5,000 target cells for sufficient consistency while providing an ideal system for answering additional questions through co-staining with additional antibodies (126).

### Antibody-Dependent Phagocytosis

Antibody-dependent phagocytosis is the least studied of the four known rituximab effector mechanisms. It is facilitated by macrophage recognition of bound rituximab through various Fcγ receptors (Figure 4, bottom right). *In vitro* measurement of ADP carries the same challenge as ADCC, but phagocytosis can be observed in real time. Microscopy and flow cytometry-based methods are most commonly relied on to quantify the amount of opsonized cancer cells that are phagocytosed. Although no *in vivo* evidence of rituximab-mediated ADP in humans exists, some evidence of ADP in knockout mouse models has been demonstrated based on a reliance on macrophage-specific FcγRIV to achieve rituximab anti-cancer effect (115).

### Trogocytosis

Trogocytosis is not thought to be a mechanism of rituximab-mediated cell death, but rather a response that occurs when other mechanisms have become exhausted and that may contribute to the reduced efficacy of rituximab. Trogocytosis, also referred to as shaving, is a process by which monocytes, neutrophils, or macrophages remove rituximab bound to CD20 by transferring plasma membrane, which has unknown contributions to rituximab resistance through an Fc receptor-mediated response (128, 129). Importantly, although trogocytosis is potentially helping cancer cells escape from mAb therapies, there is also evidence that macrophage-mediated trogocytosis can lead to target cell death rather than escape (130). These findings suggest the interplay between the immune effector-mediated responses to rituximab may be more complex than is currently known.

### Rituximab Resistance

As mentioned above, SNPs affecting the Fc receptor of NK cells have been correlated with survival. Other innate rituximab resistance mechanisms have been identified for CDC, for example, CD55 and CD59 (membrane complement-regulator proteins which prevent insertion of the MAC) are known to be expressed on some resistant lymphoma cells and reduction of those proteins *in vitro* overcomes that resistance (118). In addition, one study utilizing sera collected from CLL patients demonstrated patients were frequently deficient in C1q, C3, and C4 complement proteins and that their sera were more readily exhausted of complement activity following anti-CD20 mAb treatment, resulting in lowered CDC activity (119). In an effort to determine mechanisms of resistance to rituximab, Czuczman et al. exposed CD20 expressing lymphoma cell lines to escalating doses of rituximab exclusive of any immune effectors. From these studies, a global decrease in CD20 through pre- and post-transcriptional controls occurred in the resistant lines (131). Similarly, Small et al. observed reduction of CD20 in the sublines with acquired rituximab resistance, emphasizing antigen expression as a key mechanism of resistance (132). Reduction in pro-apoptotic factors Bax and Bak were also observed following chronic *in vitro* exposure to rituximab, which highlights potential therapies to re-sensitize resistant cells (133). Efforts are being made to circumvent resistance, either through sensitizing resistant cells or developing combination therapeutics that synergize with rituximab.

### Synergy Between Rituximab and Conventional Therapeutics

Very is little is known about how rituximab and CHOP interact *in vivo*, and this has not yet been well-studied *in vitro*. Still, there is evidence that rituximab and at least some cytotoxic chemotherapeutics have synergistic mechanisms mediating anti-cancer effects *in vitro* (27). For instance, rituximab downregulates anti-apoptosis factor Bcl-xL and sensitizes some B-cell cancers to drugs that induce cell death through cytotoxic mechanisms, thus creating synergistic effects (133, 134). CD20 binding by rituximab is also reported to increase uptake of other antibody–drug conjugates (135). Although radiation primarily functions through induction of DNA damage, there is evidence that it also recruits an immune response that may synergize with mAb therapy (136). Furthermore, DNA damage itself promotes ADCC. Fine et al. found that loss of Clr-b expression in cells under chemotherapeutic-induced genotoxic stress allowed attack by NK cells expressing NKR-P1B, which usually prevents killing of self through recognition of Clr-b on the target cell (137).

## Potential Biomarkers

Rituximab has been in use for more than 20 years, benefiting ~15% additional DLBCL patients compared with CHOP alone, and around 50% of patients when given as a monotherapy. Despite its widespread use and variable benefits, we continue to lack biomarkers to predict or measure rituximab response beyond CD20 expression and tumor burden, although the search for additional biomarkers of response is ongoing.

One type of candidate for such a predictive biomarker are SNPs in the Fc receptor genes which code for the proteins that recognize bound rituximab. These have been interrogated in several studies and may have a clinically relevant impact on rituximab efficacy, although reported conclusions are variable (138). Most reports indicate that FcγRIIIa–V158F has a poorer response compared with homozygous valine genotypes among adult patients. Indeed, a study by Weng et al. consisting of 139 FL patients showed that homozygous V/V genotypes and humoral immune response to immunoglobulin idiotype vaccines were both independent positive predictors for PFS (139). It is worth nothing that a small study including adolescents and children with mature B-cell lymphoma or leukemia, reported in 2016 by Burkhardt et al. found a response rate of 59% in children with homozygous FcγRIIIa-V158F SNP, but only 32% among patients with the major allele coding for valine (140). A recent meta-analysis of publications from searches in the PubMed and EMBASE databases up to July 2014 concluded FcγRIIa-H131R SNP, but not FcγRIIIa-V158F, is associated with inferior response to rituximab (141). Both SNPs have been implicated to affect the ability of the receptor to bind to rituximab in various studies, and the variable data on their effects on clinical response likely reflect the complicated nature of rituximab’s effect *in vivo* (115). It is possible that the complexity of the immune effector response mediated by rituximab confounds attempts at confirming a direct variable that modulates only one portion of the response. This may be why, despite better binding of obinutuzumab due to fucosylation designed to overcome decreased binding Fc-binding affinity due to the FcγRIIIa-V158F SNP, the improvement in clinical outcomes are not as dramatic as expected.

While glycoengineering of anti-CD20 is thought to improve response, variation in glycosylation of the FcγR may also be important for response. Recent findings based on *in vitro* results show that FcγRs also have glycosylation variation, and the effect of those differences is not well studied with respect to rituximab-mediated ADCC assays. Recent findings provide evidence that FcγR glycosylation has a significant impact on binding kinetics with rituximab (142). While the potential effects on ADCC were not investigated, it suggests that there are more factors that modulate binding beyond mAb fucosylation and FcγR SNPs (142).

There is some evidence that SNPs affecting CDC can predict rituximab response as well, either by direct effects on CDC or indirectly by interfering with ADCC. Indeed, in a retrospective study, a homozygous A SNP in C1qA_276_ was also correlated with improved OS in patients with DLBCL treated with R-CHOP (143). Because the polymorphism is a synonymous SNP, the effector mechanism is unclear and requires further validation. Studies looking at the epistatic or combinatorial effects of the SNPs that affect various methods of rituximab-mediated killing may also be useful for determining their *in vivo* roles. A recent study found that a SNP that correlated with reduced expression of complement-regulatory proteins such as CFHR1 and CFHR3 was associated with patient outcome (144). Interestingly, the effect appeared to vary based on the specific anti-CD20 used (144).

A comprehensive review by Di Rocco et al. enumerates numerous molecular markers for DLBCL that are associated with prognosis and response to current therapies and could be used as biomarkers for personalized medicine (145). However, few predictive biomarkers for identifying which specific patients will benefit from rituximab are reported (145). In a unique approach to identifying biomarkers, researchers performed a screen of 1,140 paired potential biomarkers in FL patients to determine if any pairs could be used to predict outcomes and thus advise new patient treatments. One pair from their screen, low CD68 expression presenting in combination with a G/G or C/G SNP in the PSMB1 gene was associated increased PFS of patients treated with bortezomib and rituximab compared with rituximab alone. A similar approach could also be used to identify patients who would benefit from rituximab monotherapy alone (146).

Because germline genetic markers can be easily probed with current technologies, they remain the most attractive potential biomarkers to facilitate personalized medicine choices. However, somatic mutations that arise in cancer tend to make more accurate predictions, although limitations such as biopsy requirements and tumor heterogeneity as well as distinguishing driver and passenger mutations, still need to be fully overcome (147). TP53 mutations are the most common *de novo* mutation in nearly all cancer types and are also common in lymphomas. TP53 is considered the master regulator of the DNA damage response and defects in this gene can cause tumors to be more resistant to the genotoxic chemotherapeutics which are a key part of most lymphoma treatments. A retrospective study evaluating data from the RICOVER-60 trial found that TP53 mutations occurred in 23.85% of the patients in the study and were independent predictors of patient survival (148). These findings highlight the need for studies able to analyze multiple key biomarkers at once, as focusing on only one could reduce significance and result in false negatives. It is known that TP53 is still a valuable prognostic marker in the post-rituximab era, but it is still unknown what role these mutations may have on rituximab efficacy specifically (149). Overexpression BCL2 is also known to be a biomarker of poor prognosis in DLBCL and is also a key factor of the rituximab direct killing pathway, although its effect on rituximab monotherapy outcomes has also not been tested (150).

Markers to monitor actual response, rather than predict response, are even more lacking. One study concluded that degranulation of NK cells following mAb treatment might be a marker of response, while granzyme B release levels was suggested in a trastuzumab study (151, 152).

The degree of CD20 expression levels among DLBCL may also be correlated to overall patient survival. It is variable both between patients and heterogeneous within an individual’s malignancy. Johnson et al. reported that a lower overall expression of CD20 is correlated with reduced survival, based on a retrospective study of DLBCL patients treated with CHOP (*n* = 82) or R-CHOP (*n* = 181). They found individuals with the low CD20 expressing disease had a median OS of 1.2 (CHOP) and 3 (R-CHOP) years, while patients with higher CD20 expression did not reach median survival in either treatment group (153).

## Biosimilars

As the first therapeutic mAb in oncology, rituximab is also one of the first to encounter competition from biosimilar products as its patent expires. The recent patent expiration (2013 and 2016 in Europe and the US, respectively), and the economic significance of rituximab as the top-selling oncology drug has spurred the development of a multitude of rituximab biosimilars. Biosimilar regulatory approval pathways have been established in both the US and Europe, offering a pathway to marketing approval designed to decrease price and increase drug accessibility while maintaining safety and efficacy standards. Increased availability of biosimilars will drive prices down, provide better accessibility to anti-CD20 mAbs worldwide, and stimulate further research that may lead to better and more widespread treatment options (154). Current pricing for rituximab biosimilars worldwide is often less than half the price of rituximab (Table 1). In the US, use of biosimilars is expected to bring a savings of $9–12 billion to the Medicare system in the next decade (155). In Table 1, we provide a summary of anti-CD20 biosimilars emerging into the marketplace; most are still in clinical trials or pending approval. However, evaluation of these biosimilars for equivalence to rituximab raises new challenges.

**Table 1:** List of rituximab biosimilars around the world including the manufacturer and their corporate location, clinical trial status and for respective disease, status, and cost relative to the rituximab.

Biosimilar (reference)	Manufacturer	Clinical trials ongoing or completed	Disease	Status	Relative^a^ cost to rituximab; $3,693 (500 mg) (191)
1B8 (192, 193)	Center of Molecular Immunology (Cuba)	Phase I	DLCBL	Pharmacokinetics and Safety in Progress	N/A
ABP 798 (194, 195)	Amgen (USA)	Phase III	NHL	Recruiting	N/A
BCD-020 (Acellbia) (173, 196–198)	Biocad (Russia)	Approved	INHL	Launched	72% less
BI 695500 (167, 168, 170–175, 199)	Boehringer Ingelheim (Germany)	Phase III	LTBFL	Terminated	N/A
CT-P10 (Truxima) (177, 200)	Celltrion (South Korea)	Approved	ASFL	Launched	72% less
GP2013 (Rixathon) (201)	Novartis Pharmaceuticals (Switzerland)	Phase III	ASFL	In progress	N/A
HLX01 (182)	Shanghai Henlius Biotech (China)	Phase III	DLBCL	In progress	N/A
JHL1101 (202, 203)	JHL Biotech (Taiwan) and Sanofi (France)	Phase I and III	NHL	In progress	N/A
Kikuzubam (204, 205)	Probiomed (Mexico)	Phase I	NHL	Withdrawn	N/A
Maball (206, 207)	Hetero (India)	Approved	CLL, DLCBL, and FL	Launched	87% less
MabionCD20 (208)	Mabion SA (Poland)	Phase III	DLBCL	Recruiting	N/A
MabTas (209–211)	Intas Pharmaceuticals (India)	Approved	NHL	Launched	76% less
MK8808 (212)	Merck Sharp & Dohme Corp. (EU)	Phase I	FL	Terminated	N/A
Novex (213, 214)	Laboratorio Elea (Argentina)	Approved	NHL	Launched	9% less
PF-05280586 (215)	Pfizer (USA)	Phase III	LTBFL	Recruiting	N/A
Reditux (155, 187, 216)	Dr. Reddy’s Laboratories (India)	Approved	DLBCL	Launched	50% less
Rituxirel (217, 218)	Reliance Life Sciences, Torrent Pharma (India)	Approved	NHL (DLBCL and FL)	Launched	84% less
RTXM83 (219)	mAbxience (Switzerland)	Phase III	DLBCL	Completed	N/A
SAIT101 (220)	Samsung BioLogics (South Korea) and AstraZeneca (UK)	Phase III	LTBFL	Completed	N/A
TL011 (221)	Teva Pharmaceuticals (Israel)	Phase III	DLBCL	Terminated	N/A
Zytux (Ristova) (222, 223)	AryoGen Biopharma (Iran)	Approved	NHL	Launched	50% less

*ASFL, advanced stage follicular lymphoma; CLL, chronic lymphocytic leukemia; DLBCL, diffuse large B-cell lymphoma; FL, follicular lymphoma; INHL, indolent non-Hodgkin lymphoma; LTBFL, low tumor burden follicular lymphoma; NHL, non-Hodgkin lymphoma; EU, European Union*.

*^a^Prices vary depending on the market and the country where the product is sold. N/A, not available*.

**Figure 5:**
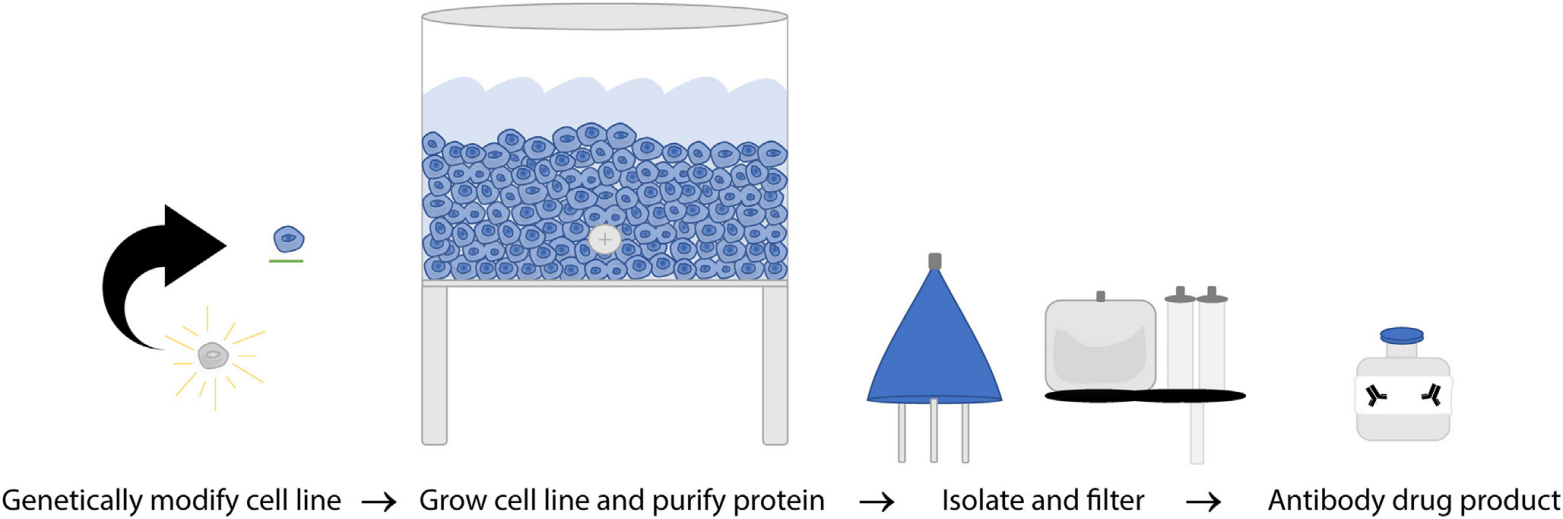
A simplified overview of rituximab manufacturing process. Once hybridoma cell lines are established from a single clone, the cultures are expanded to produce a single specific monoclonal antibody (mAb) on a massive scale. That mAb is then collected, purified, analyzed, and certified on a per lot basis.

The FDA and European Union (EU) have subtly different definitions of biosimilars but share the concept that they must be biological therapeutics that are highly similar to the original product in structure and function. WHO established guidelines in 2016 which include a few current shortfalls in assays used to compare mAb biosimilars due to variation between target and effector cells used to evaluate response as well as the challenges of reproducing results in different laboratories (156). Structural evaluation of amino acid sequences and higher order structure, as well as glycosylation state are all evaluated to ensure they are identical while functional assays including binding affinity, cell killing efficacy of *in vitro* CDC and ADCC separately, and direct killing are all evaluated as part of the path to being granted biosimilar approval (156, 157). Exact replication of a biological product is impossible, but biosimilars are designed to be as close as possible to the parent molecule. The process of making nearly identical biosimilars can be affected by two main factors: variability in the biological processes involved in manufacturing and variability in the details of the manufacturing procedures themselves. Unlike generic drugs, antibody production depends on a biological process, introducing more variables that can affect the final product. Second, producing mAbs is a proprietary process and companies do not share all manufacturing practices meaning each biosimilar company has to develop independent best practices, standard protocols, raw material sources, and equipment to utilize (158). Because of these variable factors in biosimilar production (Figure 5), it is essential to validate that the new mAb produced has the same efficacy as the original, but the protocols for doing so are hindered due to incomplete knowledge of *in vivo* effectors of rituximab response. The current requirements for the regulatory approval pathway are outlined in Table 2.

**Table 2:** Biosimilars and their respective approved regulatory standards.

Rituximab biosimilar	Approved regulator standards	Reference
BCD-020	Ministry of the Russian Federation, Department of Biotechnology and the Central Drugs Standard Control Organization (under review)	(173, 224)
CT-P10	European Medicines Agency, Korean Ministry of Food and Drug Safety, & FDA (under review)	(225, 226)
Maball	Department of Biotechnology and the Central Drugs Standard Control Organization	(227)
MabTas	*Central Drugs Standard Control Organization*	(210)
Novex	National Drugs, Foods and Medical Technology Administration (ANMAT)	(228)
Reditux	Department of Biotechnology and the Central Drugs Standard Control Organization	(210)
Rituxirel	Department of Biotechnology and the Central Drugs Standard Control Organization	(210)
Zytux	Food and Drug Organization	(229)

Although biosimilars emulate the parent antibody’s function and clinical effects in small patient trials, they are not an identical replicate for the reasons described above (154). Several initial analytical tests are used to compare biosimilars to their originator product (159). Initially, the amino acid sequence can be compared to assure identity. Other factors to be assessed are homogeneity, glycosylation state, and antibody binding to the correct antigen. SDS-PAGE characterizes homogeneity, mass spectrometry is used to determine the glycoform patterns, and the antibody crystal structure is utilized to verify binding to CD20 (159–162). In addition, there are different functional tests to assess rituximab-mediated cell death *in vitro*. As mentioned above, rituximab can induce cell death by CDC, direct apoptosis through direct signaling, and antibody-dependent cellular cytotoxicity (ADCC), as well as ADP (163–165). Biosimilar developers can confirm their product has the same effect for each mechanism *in vitro*, although no comprehensive test to evaluate interactions of effector mechanisms, which might be more representative of the *in vivo* situation, has been developed.

Once the antibody is determined to be highly similar to the parent antibody based on molecular characteristics, the effectiveness of the biosimilar is tested in small clinical trials. Unlike for their original predecessor, it is not necessary for biosimilars to go through full clinical trials to compare the efficacy in a relevant patient population (166, 167). Therefore, phase I and phase III non-inferiority trials are conducted to ensure safety, equivalent potency, and non-inferior efficacy. Post-marketing surveillance is also required by some regulatory agencies, to ensure there is no increased rate of immunogenicity (159).

The level of scrutiny a biosimilar receives is dependent mainly on the regulatory standards of the country in which it is being marketed. The rigor and standards for comparability with the originator product (in this case, rituximab) may differ depending on the approval guidelines followed (e.g., FDA, European Medicines Agency—EMA, or others). Rituximab biosimilars are produced all over the world (Table 1), and manufacturing standards are location dependent.

### Nomenclature

The development of biosimilars created a need to develop a new nomenclature. The purpose is to serve as a means of distinguishing drugs so that users know they are getting a drug that is not identical to rituximab. The typical method of drug naming through the International Nonproprietary Names is not utilized for biosimilars (168). There is currently no universal global naming system for biosimilars, but standards and drafts to establish this have been initiated. Methods of distinction include, but are not limited to adding a prefix, suffix, or color to the label (169).

### Less Financial Risk

Biosimilars provide an opportunity for less expensive therapeutic development (Table 3). Bringing a novel drug to market is rapidly increasing in cost, and currently costs more than a billion dollars (170). Biosimilars generally require smaller and fewer clinical trials, and therefore pose a lesser financial risk with a shorter timeline to approval. This is especially favorable for countries that have limited access to the originator compounds or have product shortages. With the rituximab patent expired, biotechnology and pharmaceutical companies are now legally able to participate in an 8-billion-dollar per year niche market that does not require expensive, high-risk *de novo* drug creation (171). Rituximab biosimilars have thus become an appealing development opportunity for companies in countries such as India and South Korea (172).

**Table 3:** Comparison of rituximab and biosimilars: years, phases of research, estimated costs, and market.

Considerations	Rituximab	Biosimilars
Time (years) (170, 230)	7–12	3–5
Phases of research (231)	Discovery, development, preclinical, and clinical trial phases I–III consecutive	Development, preclinical, and phase I and III
Estimated cost (232)	1 billion	100 million
Total market (233)	85.4 billion	

### BCD-020

BCD-020 is a biosimilar with the trade name of AcellBia. It is the first mAb biosimilar developed in Russia (173). Data reportedly suggest BCD-020 is comparable to the parent drug with regard to PK/pharmacodynamics (PD), safety, and efficacy. However, these results and those regarding the clinical studies are not publicly available (174). Regardless of the lack of transparency, biosimilar production companies are emerging and increasing competition on a global scale. BCD-020 development has created increased competition between biotech companies in Russia (Biocad) and the US (Genentech/Roche). Although Russia is less established in the biotechnology market, they have a financial advantage, with a highly educated workforce and low employment costs relative to the US (175). Competition such as this may cause the price of parent and biosimilar products to decrease, although regulatory standards ensuring a high-quality biosimilar product must also be considered.

### CT-P10

Also known as Truxima™, this is the first biosimilar to be granted marketing authorization by the EU, in 2016 (159). A phase I and phase III trial of CT-P10 was done to confirm safety, similar PK/PD, and efficacy in RA patients (176). There were no significant differences between CT-P10 and rituximab, and CT-P10 was also tested in untreated advanced stage FL patients. Patients were randomized to either R-CVP (*n* = 70) or CT-P10-CVP (*n* = 70) for eight cycles, and the primary endpoint was response rate. PK/PD was also monitored in a subset of patients and safety was assessed in all patients (177). The ORR was 97.3% in the CT-P10-CVP group and 92.6% in the R-CVP group, meeting the endpoint for non-inferiority (177). PK/PD and safety measures were also similar between the two groups (177). These studies led to the approval to market CT-P10 by the EMA for all rituximab indications. It is important to note that extrapolation of treatment indications beyond the tested patient populations is permissible by the EMA and FDA, based on the totality of the data and the diversity of disease populations tested in clinical trials used for the approval application. This is likely why one autoimmune and one oncologic disease population were studied in CT-P10 clinical trials. Application for FDA approval of CT-P10 has been submitted and is pending.

### GP2013

Also known as Rixathon™, this biosimilar is also approved for use in the EU, and is the second anti-CD20 biosimilar for which an FDA application has been submitted in the US (along with CT-P10, above). Clinical studies have included a PK/PD study in RA, a phase III study in RA (178), and a confirmatory safety and efficacy phase III study in FL (179). In the ASSIST-FL study, 629 untreated, advanced FL patients were randomly assigned to either R-CVP or GP2013-CVP for eight cycles, followed by 2 years of monotherapy mAb maintenance in responders. ORR, the primary endpoint, was 87% with GP2013 and 88% with rituximab. Safety profiles were similar in the two groups as well. It is noteworthy that both GP2013 and CT-P10 were approved without PFS efficacy results being reported, indicating that response rate is a sufficient surrogate endpoint in rituximab biosimilar studies.

### HLX01

HLX01 is the biosimilar closest to approval in China and has been tested in clinical trials both in DLBCL and in severe RA. The first clinical trial in 2015 determined the PK and PD of this biosimilar relative to rituximab (180). Afterward, the effects of HLX01 and rituximab were compared in patients with CD20-positive B-cell lymphomas (181). In 2016, CHOP with HXL01 was compared with CHOP and rituximab in DLBCL patients, to ensure similar efficacy (182). Lastly, a fourth clinical trial in phase I/II testing the efficacy of HLX01 in patients with severe RA is scheduled to be completed in 2018 (183).

### Reditux

This is the world’s first biosimilar and was launched in 2007, before the rituximab patent expiration date (184). Like Russian biosimilar companies, India is also contributing to affordable pricing and reduced dependence on foreign imports in their country by producing their own biosimilars. The combination of the WHO publishing standards for the biosimilar evaluations (185) and the need for studies on post marketed products (186) led to a retrospective study in 2013. Response rates, toxicity, progression-free survival, and overall survival for 173 DLBCL patients (101 treated with R-CHOP; 72 treated with Reditux-CHOP) were compared, and were similar in all respects (187). In 2016, another study was reported assessing the PK in 21 DLBCL patients treated with Reditux-CHOP, and results suggested that Reditux has a similar PK relative to rituximab (188). However, data from that study demonstrated a decrease in the estimated central volume of distribution relative to rituximab by 68–76% (189). Tout et al. hypothesized there could be one of two reasons for this: either there was an alteration due to differences in tumor burden or to a dissimilarity in the methods used to compared PK in rituximab and Reditux. Further prospective studies will likely be required to establish equivalent potency and efficacy prior to approval in the US or Europe, but Reditux is already increasing accessibility in Asia, Latin America, and the Middle East (155).

Many other rituximab biosimilars, including BI 695500, Kikuzubam, SAIT101, and TL011, halted development prematurely due to either changes in regulatory standards, strategic marketing decisions, and/or the health of the economy (Table 1).

### Transparency

There is a lack of public information available for some of the biosimilars listed above, particularly regarding how data is collected, analyzed, and compared with rituximab. Increasing the transparency of biosimilar development may help support the overall claim that these biosimilars are equivalent in efficacy to rituximab while still being a cheaper treatment option. Given that there are biosimilars produced all over the world, it would be helpful if international regulatory standards be aligned as much as possible. More universal biosimilar drug development and approval processes may result in further decreasing the price of biosimilar mAbs by increasing global access to them, along with comfort in the approval process. Educating prescribing physicians about biosimilars and the approval process is another important component that will determine the level of biosimilar uptake in various markets.

## Conclusion

As the first mAb approved for oncology treatment, rituximab is an important milestone in the age of immunotherapeutics and is currently used to treat the majority of B-cell NHL as a monotherapy or in combination with conventional lymphoma therapies. Its use has substantially improved the outcome among all B-cell lymphoma patients. Rituximab has paved the way for immunotherapy biologic discovery, regulatory pathways, and clinical practices; and it is now indirectly outlining how the world deals with biosimilar development in the field of oncology.

Despite rituximab’s long history of successful application, much remains to be discovered. Like other mAb therapies, rituximab facilitates cell killing through various mechanisms including direct signaling of cell death as well as immune-mediated responses such as CDC, ADCC, and ADP. However, we do not yet know which of these mechanisms play the most significant role *in vivo*, nor do we understand why only a subset of patients achieve a durable response. Furthermore, we do not yet know the ideal dosage schedules, and many *de novo* anti-CD20’s have been approved for different dosing, which makes direct comparison more difficult. We also lack biomarkers to reliably predict which patients will benefit from rituximab, or even which patients are benefiting from its inclusion in combination therapies. These gaps in knowledge surrounding rituximab make assessing next generation anti-CD20 therapies and rituximab biosimilars a challenging goal, providing opportunities for improvement as the relative efficacies of those new mAbs are evaluated.

The field of anti-cancer immunotherapies continues to deliver powerful new treatment options beyond mAb therapies. However, the areas that are still poorly understood are being actively studied and represent a potential to improve rituximab, and possibly all mAb therapies, with the end goal of making them cheaper, more accessible, and improving their efficacy for the largest number of patients possible.

## Author Contributions

TP, CL, and KR all contributed to the writing and editing of this manuscript.

## Conflict of Interest Statement

The submitted work was carried out with no personal, professional or financial relationships that could potentially be construed as a conflict of interest.

## Abbreviations

ABC, activated B-cell-like; ADCC, antibody-dependent cell-mediated cytotoxicity; ADP, antibody-dependent phagocytosis; BCR, B-cell receptor; BL, Burkitt lymphoma; BR, rituximab with bendamustine; CAR, chimeric antigen receptor; CLL, chronic lymphocytic leukemia; CDC, complement-dependent cytotoxicity; CR, complete remission; Cr, chromium; DLBCL, diffuse large B-cell lymphoma; EFS, event-free survival; EU, European Union; FCR, fludarabine, cyclophosphamide, and rituximab; FDA, Food and Drug Administration; FFS, failure-free survival; FL, follicular lymphoma; GCB, germinal center B-cell-like; HCL, hairy cell leukemia; HIS, human immune system; INN, International nonproprietary names; IR, ibrutinib plus rituximab; LPL, lymphoplasmacytic lymphoma; mAb, monoclonal antibody; MAC, membrane attack complex; MALT, mucosa-associated lymphoid tissue; MCL, mantle cell lymphoma; MR, maintenance rituximab; MZL, marginal zone lymphoma; NHLs, non-Hodgkin lymphomas; NK, natural killer; OB, ofatumumab with bendamustine; OKT3, muromonab-CD3; ORR, overall response rate; PD, pharmacodynamics; PK, pharmacokinetics; PMBCL, primary mediastinal large B-cell lymphoma; R-CHOP, rituximab, cyclophosphamide, doxorubicin, vincristine, and prednisone; SLL, small lymphocytic lymphoma; WHO, World Health Organization; WM, Waldenstrom macroglobulinemia.
